# Exposures of 129 Preschool Children to Organochlorines, Organophosphates, Pyrethroids, and Acid Herbicides at Their Homes and Daycares in North Carolina

**DOI:** 10.3390/ijerph110403743

**Published:** 2014-04-03

**Authors:** Marsha K. Morgan, Nancy K. Wilson, Jane C. Chuang

**Affiliations:** 1 National Exposure Research Laboratory, United States Environmental Protection Agency,109 T.W. Alexander Drive, Research Triangle Park, NC 27709, USA; 2 Battelle, Durham, NC 27713, USA; E-Mail: njkwilson@nc.rr.com; 3 Battelle, Columbus, OH 43201, USA; E-Mail: ccjane20@hotmail.com

**Keywords:** children, pesticides, exposure, intake dose, residences, daycare centers

## Abstract

Few data exist on the concurrent exposures of young children to past-use and current-use pesticides in their everyday environments. In this further analysis of study data, we quantified the potential exposures and intake doses of 129 preschool children, ages 20 to 66 months, to 16 pesticides (eight organochlorines, two organophosphates, three pyrethroids, and three acid herbicides). Environmental samples (soil, dust, outdoor air, and indoor air) and personal samples (hand wipes, solid food, and liquid food) were collected at 129 homes and 13 daycare centers in six counties in North Carolina between 2000 and 2001. *α*-Chlordane, *γ*-chlordane, heptachlor, chlorpyrifos, diazinon, *cis-*permethrin, *trans*-permethrin, and 2,4-dichlorophenoxyacetic acid (2,4-D) were detected ≥50% in two or more media in both settings. Of these pesticides, the children’s estimated median potential intake doses through dietary ingestion, nondietary ingestion, and inhalation routes were the highest for 2,4-D and *cis/trans*-permethrin (both 4.84 ng/kg/day), *cis/trans*-permethrin (2.39 ng/kg/day), and heptachlor (1.71 ng/kg/day), respectively. The children’s estimated median potential aggregate intake doses by all three routes were quantifiable for chlorpyrifos (4.6 ng/kg/day), *cis/trans*-permethrin (12.5 ng/kg/day), and 2,4-D (4.9 ng/kg/day). In conclusion, these children were likely exposed daily to several pesticides from several sources and routes at their homes and daycares.

## 1. Introduction

Since the 1950s, a number of commercial products containing pesticides have been used to kill insects and weeds in residential and agricultural settings in the United States (U.S.). Some of the major classes of insecticides that have been applied to control insects in these settings include the organochlorine (OC), organophosphorus (OP), and pyrethroid (PY) insecticides. For the OC insecticides (*i.e*., aldrin, chlordane, DDT, dieldrin, endrin, heptachlor, and lindane), the U.S. Environmental Protection Agency (U.S. EPA) has phased-out almost all uses since the late-1980s because they are persistent and bioaccumulative in the body [1,2,3,4,5,6]. The OP insecticides, particularly chlorpyrifos and diazinon, are still commonly applied on agricultural crops. However, the U.S. EPA phased-out almost all residential and other similar uses of chlorpyrifos and diazinon at the end of 2001 and 2004, respectively, to reduce children’s exposures and potential health risks [7,8]. The PY insecticides (e.g., permethrin and cyfluthrin) have replaced many of the residential uses of the OP insecticides, and they are also extensively applied on agricultural fields [9]. Lastly, one of the major classes of herbicides widely used to kill unwanted weeds on lawns, pastures, and croplands have been the acid (AC) herbicides, and frequently applied ones today include dicamba and 2,4-D [10,11,12,13]. 

Only a few published studies have reported concurrent levels of the OC, OP, and PY insecticides and the AC herbicides in several media at children’s homes and daycare centers in the U.S. [14,15]. Wilson *et al*. [14] reported measureable concentrations of aldrin, *α-*chlordane, *γ*-chlordane, *p,p’*-DDT, dieldrin, endrin, heptachlor, lindane, chlorpyrifos, diazinon, and 2,4-D in multimedia samples collected at 10 child daycare centers in North Carolina (NC) in the spring 1997. In a proceeding study, Wilson *et al*. [15] also showed measureable levels of these same 11 pesticides in multimedia samples collected at the homes and daycare centers of nine preschool children in NC in the summer of 1997. In the Wilson *et al*. [15] study, the children’s estimated median potential aggregate intake doses to these pesticides ranged from 0.15 ng/kg/day (endrin) to 87.6 ng/kg/day (2,4-D). This research suggests that young children are likely being exposed to several pesticides, including past-use ones, on a daily basis in their everyday environments.

In 1999, the U.S. EPA designed the Children’s Total Exposure to Persistent Pesticides and Other Persistent Organic Pollutants (CTEPP) study in part to fill critical data gaps on young children’s exposures to pesticides in direct response to the Food Quality Protection Act (FQPA) of 1996 [16,17]. The FQPA of 1996 specifically mandated that the US EPA consider the aggregate exposures and cumulative health risks of infants and children before setting pesticide tolerances in food [16]. The CTEPP study was built upon the data and information obtained in the earlier pilot studies conducted by Wilson *et al*. [14,15]. The CTEPP study is the first large-scale study in the U.S. to quantitatively assess preschool children’s exposures to a number of pesticides, including past-use one, from several sources and routes of exposure [17]. It investigated the exposures of 256 preschool children (ages 20–67 months) to over 40 chemicals, including pesticides, commonly found at their homes and daycare centers in North Carolina (NC, U.S.) and Ohio (OH, U.S.). 

In previous publications [18,19,20,21], we examined separately the CTEPP children’s potential exposures and potential intake doses to four current-use pesticides (*i.e*., chlorpyrifos, diazinon (*OH*, *only*), 2,4-D, and/or permethrin (*OH*, *only*)) in media at their homes and/or daycare centers in NC and OH. In this present work, we conducted a further analysis of the study data that investigated the CTEPP children’s concurrent exposures to nine past-use pesticides (aldrin, *α-chlordane*, *γ*-chlordane, *p,p’*-DDT, dieldrin, endrin, heptachlor, lindane, 2,4,5-trichlorophenoxyacetic acid (2,4,5-T)) and seven current-use pesticides (chlorpyrifos, diazinon, cyfluthrin, *cis*-permethrin, *trans*-permethrin, dicamba, and 2,4-D) in media at their homes and daycare centers in NC. For this analysis, we examined the demographic data, questionnaire data, environmental measurement data, and personal measurement data for the subset of 129 children that participated in the NC component of the study. The objectives were to quantify the distributions of 16 different pesticides in several environmental and personal media for a subset of CTEPP children at their homes and daycares in NC, to estimate the children’s potential exposures and potential intake doses to the pesticides by the dietary, nondietary, and inhalation routes of exposure, and to identify the major sources and exposure routes. 

## 2. Methods

### 2.1. Study Cohort

The study design for the CTEPP study has been discussed in-depth by Wilson *et al*. [17]. Briefly, the participants were recruited from six counties in NC from July 2000 to April 2001 and from six counties in OH from April 2001 to November 2001. The study cohort consisted of 256 preschool children; 129 children participated from NC and 127 children participated from OH. In NC, 66 children stayed-at-home with their adult caregivers during the day and 63 children attended daycare during the day. In OH, 69 children stayed-at-home with their adult caregivers during the day and 58 children attended daycare during the day. For the daycare group of children, environmental and personal samples were collected at both their homes and daycare centers. Environmental samples (soil, dust, outdoor air, and indoor air) and personal samples (hand wipes, solid food, and liquid food) were collected over a 48-h monitoring period at 129 homes and 13 daycare centers in NC and at 127 homes and 16 daycare centers in OH. Field staff collected environmental samples at both locations, and adult caregivers (*i.e*., parents and daycare teachers) collected personal samples from the children. 

### 2.2. Human Subjects Review

The CTEPP study was a human observational research study, as defined in 40 Code of Federal Regulations, Part 26.402 [22]. The study protocol and procedures used to obtain informed consent of the adult caregivers and the assent of the preschool children were approved by an independent institutional review board (Battelle) before beginning the study and complied with all applicable requirements of the Common Rule regarding additional protections for children (Subpart D). The study protocol and procedures were also approved by the US EPA’s Human Subjects Research Official prior to starting the study.

### 2.3. Field Sampling

Detailed descriptions of the field sampling activities that occurred over a 48-h monitoring period at the children’s homes and/or daycare centers have been described in Wilson *et al*. [17]. The collection of the environmental samples and personal samples are briefly described below. Soil samples consisted of scraping the surface of a 0.1 m^2^ area of bare soil with a putty knife (nearest a child’s play area) and placing it into a pre-cleaned glass jar. Indoor floor dust samples were collected from a 0.76 m^2^ of carpeting from the child’s main activity area (e.g., living room or classroom) with a high volume surface sampler (Cascade Stack Sampling Systems, Bend, OR, USA) and transferring the sample to a pre-cleaned glass jar. Outdoor air was sampled over a 48-h period using a URG-2000 cartridge with a Thomas pump generating a flow rate of ~4.0 L/min. For indoor air, 48-h samples were collected using a URG-2000 cartridge with a SKC pump (~4.0 L/min) in the child’s main activity area. Hand wipe samples consisted of the adult caregivers wiping the front and back of both hands of the children with a 100 cm^2^ pre-wetted cotton pad (SOF-WICK, Johnson and Johnson, Arlington, TX, USA) with 2 mL of 75% isopropanol and placing it into a pre-cleaned glass jar. Duplicate diet samples were collected from each child by their adult caregiver over the 48-h period; solid and liquid food samples were collected separately in 2 L pre-cleaned glass jars. Solid foods included all the fruits, vegetables, meats, dairy products, and desserts eaten by each child. Liquid foods included all of the beverages, excluding drinking water, consumed by each child. Examples of typical solid foods collected were apples, bananas, carrots, lunch meats, cheeses, and cookies, and examples of typical liquid foods collected were fruit juices, milk, and soft drinks. Duplicate amounts of solid and liquid foods were collected at homes, and duplicate serving of solid and liquid foods were collected at daycares. All samples were kept at reduced temperatures in coolers until picked up by field staff.

### 2.4. Sample Analyses

Detailed extraction and analytical procedures for the target pesticides in the environmental media and personal media can be found in Morgan *et al*. [18]. Briefly, the extraction methods for the OC, OP, and PY insecticides in each medium are as follows: soil samples (2 g) and dust samples (0.5 g) were sonicated with 10% diethyl ether in hexane, “concentrated” by Kuderna-Danish (KD) evaporation, followed by Florisil solid phase extraction (SPE), and concentrated again. Air samples and wipe samples were Soxhlet-extracted (~14-h) with dichloromethane (DCM), concentrated and subjected to Florisil SPE clean-up (if needed). Solid food samples were homogenized by a food chopper, and then 12 g of food were mixed with Extrelute and extracted using accelerated solvent extraction (ASE) with DCM, concentrated, and fractionated by gel permeation chromatography (GPC) with DCM, followed by an ENVI-Carb clean up. Liquid food samples (30 mL) were refluxed with DCM, filtered, concentrated, and then GPC clean-up with DCM. All sample extracts were adjusted to 1 mL with solvent and placed into glass vials. The extraction methods for the AC herbicides in each medium are as follows: soil samples (5 g) were mixed with Extrelute, extracted using ASE with acetone, and concentrated. Dust samples (0.5 g) were mixed with sand, then extracted using ASE with acetone, and concentrated. Air samples and hand wipe samples were Soxhlet-extracted with acetonitrile and concentrated. Solid food samples were homogenized with a food chopper and 8 g were mixed with extrelute and extracted using ASE with methanol, and concentrated. Liquid food samples (10 mL) were mixed with Extrelute and extracted using ASE with methanol, and concentrated. All extracts were reduced to 1 mL with solvent, derivatized with N-(*tert*-butyldimethylsilyl)-N-methyltrifluoroacetamine, and placed into glass vials. 

The surrogate recovery standard used for the OC, OP, and PY insecticides was *p,p’*-DDE-d_4_. The internal standards for the three classes of insecticides were phenanthracene-d_10_/*p,p’*-dibromobiphenyl, diazinon-d_10_, and *p,p’*-dibromobiphenyl, respectively. The surrogate recovery standard for the AC herbicides was 2,4-D-C_13_, and the internal standard was dicamba-d_3_. Matrix spikes were used for each target pesticide in all sampled media. All extracts were analyzed by a gas chromatograph with mass selective detection (6890/5973A Hewlett-Packard) in the selected ion monitoring (SIM) mode. Table 1 provides the estimated limits of detection (LODs) for the target pesticides in the environmental media and personal media. The estimated limit of detection (LOD) was defined “as the minimum analyte level detected in a sample (in a given medium) and was estimated to be one-half the limit of quantification (LOQ)” [23]. The estimated LOQ was about twice the LOD for each pesticide by matrix.

### 2.5. Quality Assurance and Quality Control

Field blanks for the pesticides were below the LODs in all media, except for chlorpyrifos and *cis*-permethrin in only 1 of 12 air samples each. The mean values of these two insecticides were below the LODs in the air samples, so no background corrections were made. Laboratory blanks for all pesticides were below the LODs in each sampled media. Relative percent differences between duplicate samples (aliquots of the same sample) for the target pesticides were less than 10% in all media, except for chlorpyrifos in the air samples (24%) and dust/soil samples (14%). Relative percent differences between the analytical duplicates (aliquots of the same sample extract) for the target pesticides were less than 8% in the sampled media. The mean recoveries for the surrogate recovery standards, *p,p’*-DDE-d_4_ and 2,4-D-C_13_, were between 73% and 100% and 75% and 91%, respectively, in all media. The matrix spikes for the OC, OP, and PY insecticides had mean recoveries in media from 71%–130%, except for diazinon (54%) and cyfluthrin (64%) in the liquid food samples and diazinon (58%) in the solid food samples. For the AC herbicides, the matrix spikes had mean recoveries in all media between 72% and 99%, except for the indoor and outdoor air samples (64%–69%).

### 2.6. Statistical Analyses

Data values below the LOD were assigned the value of the LOD divided by the square root of two, except for the liquid food concentration data. Since the pesticide concentrations in the liquid food samples were barely detectable on the gas chromatographs, a more conservative value of LOD divided by the square root of 10 was used [18]. Descriptive statistics (frequency of detection, percentiles (50th and 95th), and range) were computed for the pesticides in each medium at both the homes and daycare centers.

**Table 1:** Estimated limits of detection (LODs) for the target pesticides in environmental and personal media **^a^.**

Class ^b^	Pesticide	Environmental				Personal		
		Soil (ng/g)	Dust (ng/g)	Outdoor Air (ng/m^3^)	Indoor Air (ng/m^3^)	Hand Wipe (ng/cm^2^)	Solid Food (ng/g)	Liquid Food (ng/mL)
OC	Aldrin	0.49	2.0	0.09	0.09	0.003	0.08	0.03
OC	*α*-Chlordane	0.49	2.0	0.09	0.09	0.003	0.08	0.03
OC	*γ*-Chlordane	0.49	2.0	0.09	0.09	0.003	0.08	0.03
OC	*p,p’*-DDT	0.49	2.0	0.09	0.09	0.003	0.08	0.03
OC	Dieldrin	0.49	2.0	0.09	0.09	0.003	0.08	0.03
OC	Endrin	0.49	2.0	0.09	0.09	0.003	0.08	0.03
OC	Heptachlor	0.49	2.0	0.09	0.09	0.003	0.08	0.03
OC	Lindane	0.49	2.0	0.09	0.09	0.003	0.08	0.03
OP	Chlorpyrifos	0.49	2.0	0.09	0.09	0.003	0.08	0.03
OP	Diazinon	0.49	2.0	0.09	0.09	0.003	0.08	0.03
PY	Cyfluthrin	4.9	20	0.87	0.87	0.03	0.83	0.33
PY	*cis*-Permethrin	0.49	2.0	0.09	0.09	0.003	0.08	0.03
PY	*trans*-Permethrin	0.49	2.0	0.09	0.09	0.003	0.08	0.03
AC	Dicamba	0.40	4.0	0.17	0.17	0.01	0.25	0.20
AC	2,4-D	0.40	4.0	0.17	0.17	0.01	0.25	0.20
AC	2,4,5-T	0.40	4.0	0.17	0.17	0.01	0.25	0.20

Notes: **^a^** The estimated limit of quantification (LOQ) was about twice the reported LOD for a pesticide in each sample medium; **^b^** Pesticide classes include organochlorine insecticides (OC), organophosphorus insecticides (OP), pyrethroid insecticides (PY), and AC herbicides.

**Table 2:** Equations used to calculate the children’s estimated potential exposures to a pesticide by the dietary, nondietary, and inhalation exposure routes **^a,b^**.

Equation	Variable Definitions
**Dietary Ingestion Route**	
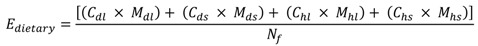	E_dietary_ = Maximum potential absorbed dose of each child over a day (ng/day)
	C_dl_ = Level of pesticide in the liquid food sample at daycare (ng/mL)
	C_hl_ = Level of pesticide in the liquid food sample at home (ng/mL)
	C_ds_ = Level of a pesticide in the solid food sample at daycare (ng/g)
	C_hs_ = Level of a pesticide in the solid food sample at home (ng/g)
	M_dl_ = Total volume of the liquid food sample at daycare (mL)
	M_hl_ = Total volume of the liquid food sample at home (mL)
	M_ds_ = Total weight of the solid food sample collected at daycare (g)
	M_hs_ = Total weight of the solid food sample collected at home (g)
	N_f_ = Number of days food samples were collected for each child (day)
**Nondietary Ingestion Route**	
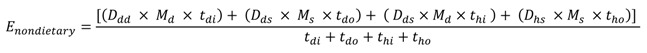	E_nondietary_ = Maximum potential absorbed dose of each child over a day (ng/day)
	D_dd_ = Level of pesticide in the dust sample at daycare (ng/g)
	D_hd_ = Level of pesticide in the dust sample at home (ng/g)
	D_ds_ = Level of pesticide in the soil sample at daycare (ng/g)
	D_hs_ = Level of pesticide in the soil sample at home (ng/g)
	t_di_ = Time spent inside at daycare (h/day)
	t_hi_ = Time spent inside at home (h/day)
	t_do_ = Time spent outside at daycare (h/day)
	t_ho_ = Time spent outside at home (h/day)
	M_d_ = Estimated dust ingestion rate (g/day)
	M_s_ = Estimated soil ingestion rate (g/day)
**Inhalation Route ^c^**	
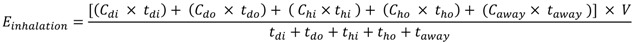	E_inhalation_ = Maximum potential absorbed dose of each child over a day (ng/day)
	C_di_ = Level of pesticide in the indoor air sample at daycare (ng/m^3^)
	C_hi_ = Level of a pesticide the indoor air sample at home (ng/m^3^)
	C_do_ = Level of a pesticide in the outdoor air sample at daycare (ng/m^3^)
	C_ho_ = Level of a pesticide in the outdoor air sample at home (ng/m^3^)
	C_away_ = Indoor air level of pesticide at places away from daycare or home (ng/m^3^)
	t_di_ = Time spent inside at daycare (h/day)
	t_hi_ = Time spent inside at home (h/day)
	t_do_ = Time spent outside at daycare (h/day)
	t_ho_ = Time spent outside at home (h/day)
	t_away_ = Time spent inside at places away from daycare or home (h/day)
	V = Estimated ventilation rate (m^3^/day)

Notes: ^**a**^ The estimated potential intake dose of a child was calculated by dividing E_dietary,_ E_nondietary_ or E_inhalation_ by their body weight (kg) and a default absorption rate of 50%; **^b^** The equations were reported earlier in Morgan *et al*. [18]; **^c^** C_away_ was calculated by using the median indoor air concentration of C_hi_ and C_di_ since air samples were not collected in locations where children spent their time away from home or daycare.

The estimated potential exposures (ng/day) of the 129 NC children were calculated for “frequently detected” pesticides through the dietary, nondietary, and inhalation routes using equations reported in a previous article [18] and are presented in Table 2. A “frequently detected” pesticide was defined here as having at least a 50% detection frequency in two or more different sampled media. There were a total of eight pesticides that met this criteria: *α*-chlordane, *γ*-chlordane, heptachlor, chlorpyrifos, diazinon, *cis*-permethrin, *trans*-permethrin, and 2,4-D. In Table 2, the children’s potential intake doses (ng/kg/day) to the frequently detected pesticides were computed by dividing E_dietary,_ E_nondietary,_ or E_inhalation_ by their body weight (kg) and by a default absorption rate of 50%. We assumed a default 50% absorption rate for a pesticide by each route of exposure as little published data exist in humans [23,24]. The dermal route for the pesticides was not quantified for these children as past research has indicated that this is a minor exposure route [14,18,19]. In addition, the children’s estimated potential aggregate exposures and potential aggregate intake doses were calculated for frequently detected pesticides (chlorpyrifos, permethrin, and 2,4-D) that had measureable levels for all three exposure routes. All statistical analyses were performed using SAS Version 8.0 (SAS, Cary, NC, USA).

## 3. Results

### 3.1. Demographic and Pesticide-use Data

In this NC cohort of CTEPP preschool children, there were a total of 58 males and 71 females. The children’s median age was 47 months, and their ages ranged between 20 months and 66 months. The racial background of the children was reported as white (55%), black (37%), Hispanic (4%), other (3%), and unknown (1%). The majority of the children (61%) lived in homes with a total household income of less than $50,000 per year. The children’s median body weight was 16.7 kg, and ranged from 10.4 to 44.1 kg.

In the questionnaires, 74% and 38% of the 129 homeowners reported applying products containing insecticides and herbicides, respectively, since residing (≥1 year) at their residences. Of these homeowners, 90% had used products that contained insecticides and 88% had used products that contained herbicides within a year of field sampling at their homes. For the 13 daycares, 62% and 31% had applied products with insecticides and herbicides, respectively, in the past at their facilities (≥1 year). Of these daycares, 88% had used products with insecticides and 100% had used products with herbicides within a year of the field sampling.

### 3.2. Pesticide Concentrations in Environmental and Personal Media

Table 3 and Table 4 present the distributions of the 16 pesticides measured in the environmental media collected at 129 homes and 13 daycare centers in NC. The OC insecticides were detected in all sampled media, except for aldrin in soil samples and outdoor air samples (daycares, only). Among the measured OC insecticides, only *α*-chlordane and *γ*-chlordane were detected ≥50% in the dust, ndoor air, and outdoor air samples at both locations.

**Table 3:** Concentrations of pesticides in environmental media collected at 129 children’s homes in North Carolina.

Pesticide	Soil (ng/g)				Dust (ng/g)				Outdoor Air (ng/m^3^)				Indoor Air (ng/m^3^)			
	%	50th	95th	Range	%	50th	95th	Range	%	50th	95th	Range	%	50th	95th	Range
*Organochlorine Insecticides*																
Aldrin	0	----	----	----	16	< **^a^**	35.4	<−276	9	<	0.27	<−2.9	38	<	9.90	<−413
*α*-Chlordane	30	<	16.2	<−2,670	95	22.0	401	<−2,010	50	0.08	1.19	<−3.74	98	0.89	24.6	<−54.7
*γ*-Chlordane	30	<	11.9	<−4,440	97	30.6	649	<−1,980	61	0.12	1.78	<−10.9	100	1.51	40.5	0.09–92.1
*p,p’*-DDT	20	<	13.3	<−544	39	<	208	<−4,080	12	<	0.32	<−2.16	37	<	3.28	<−90.2
Dieldrin	14	<	9.78	<−321	43	<	158	<−473	13	<	0.40	<−1.6	41	<	7.47	<−56.3
Endrin	4	<	<	<−5.44	19	<	118	<−317	39	<	0.95	<−1.49	34	<	1.59	<−15.1
Heptachlor	3	<	<	<−86.5	41	<	552	<−1,610	61	0.29	4.68	<−39.3	92	6.80	124	<−465
Lindane	6	<	0.68	<−60.2	14	<	51.2	<−1,000	12	<	0.42	<−6.15	13	<	7.73	<−18.5
*Organophosphorus Insecticides*																
Chlorpyrifos **^b^**	18	<	16.7	<−1,170	100	135	1,180	11.5–15,100	84	0.27	4.3	<−45.9	100	6.21	70.7	0.3–391
Diazinon	18	<	4.24	<−5,470	96	17.5	388	<−11,000	50	0.09	1.10	<−42.8	100	2.02	63.7	0.14–1,780
*Pyrethroid Insecticides*																
Cyfluthrin	12	<	32.1	<−187	48	<	1660	<−4,100	0	----	----	----	4	<	<	<−183
*cis*-Permethrin	23	<	13.4	<−1,360	100	804	21,100	67.1–311,000	16	<	0.48	<−1.62	66	0.58	7.9	<−34.4
*trans*-Permethrin	23	<	17.9	<−1,610	100	629	19,400	51.3–32,000	16	<	0.26	<−1.01	66	0.36	7.62	<−40.9
*Acid Herbicides*																
Dicamba	6	<	0.40	<−26.1	23	<	70.7	<−159	8	<	0.43	<−0.76	1	<	<	<−0.48
2,4-D **^c^**	19	<	3.28	<−30.5	66	32.3	820	<−7,390	19	<	0.76	<−2.26	46	<	3.03	<−5.88
2,4,5-T	1	<	<	<−1.12	0	----	----	----	9	<	0.49	<−1.66	7	<	0.67	<−2.12

Notes: ^**a**^ Below the limit of detection (LOD) for a pesticide; **^b^** Concentration data in environmental media at 129 NC children’s homes were previously reported in Morgan *et al*. [18]; **^c^** Concentration data in environmental media were reported for 66 out of 127 NC children’s homes in Morgan *et al*. [20].

**Table 4:** Concentrations of pesticides in environmental media collected at 13 child daycare centers in North Carolina.

Pesticide	Soil (ng/g)				Dust (ng/g)				Outdoor Air (ng/m^3^)				Indoor Air (ng/m^3^)			
	%	50th	95th	Range	%	50th	95th	Range	%	50th	95th	Range	%	50th	95th	Range
*Organochlorine Insecticides*																
Aldrin	0	----	----	----	15	< **^a^**	1,410	<−2,440	0	<	----	----	55	0.82	29.5	<−35.0
*α*-Chlordane	46	<	11.9	<−11.9	100	43.0	987	4.61–1,080	85	0.15	108	<−108	100	0.51	15.7	0.14–17.7
*γ*-Chlordane	46	<	13.1	<−13.1	100	66.6	1,210	5.57–1,210	85	0.28	115	<−115	100	0.79	42.6	0.21–47.7
*p,p’*-DDT	15	<	7.78	<−7.78	30	<	426	<−657	15	<	0.34	<−0.34	20	<	3.04	<−5.85
Dieldrin	8	<	2.49	<−2.49	58	20.3	1,730	<−1,730	23	<	0.50	<−0.50	30	<	4.81	<−4.93
Endrin	8	<	3.03	<−3.03	15	<	111	<−159	54	0.17	1.04	<−1.04	35	<	1.22	<−1.64
Heptachlor	23	<	2.03	<−2.03	55	19.4	942	<−1,040	69	0.54	54.8	<−54.8	100	5.40	284	1.4–287
Lindane	8	<	0.93	<−0.93	20	<	51.4	<−53.6	8	<	0.11	<−0.11	20	<	7.05	<−8.97
*Organophosphorus Insecticides*																
Chlorpyrifos **^b^**	7	<	<	<−0.76	100	142	921	12.4–921	77	0.34	1.53	<−1.53	100	3.0	25.3	0.58–29.4
Diazinon	0	----	----	----	100	65.2	6,880	3.06–6,880	62	0.12	0.29	<−0.29	100	2.27	70.2	0.17–106
*Pyrethroid Insecticides*																
Cyfluthrin	8	<	42.2	<−42.2	42	<	1,750	<−1750	0	----	----	----	10	<	1.60	<−1.74
*cis*-Permethrin	8	<	2.55	<−2.55	100	806	19,700	113–29,000	39	<	0.45	<−0.45	55	0.11	2.45	<−3.05
*trans*-Permethrin	8	<	2.20	<−2.20	100	856	209,00	125–29,900	39	<	0.34	<−0.34	50	<	2.14	<−2.76
*Acid Herbicides*																
Dicamba	0	----	----	----	5	<	<	<−23.6	8	<	0.21	<−0.21	0	----	----	----
2,4-D	0	----	----	----	75	23.0	77.5	<−93.7	46	<	0.66	<−0.66	60	0.33	6.17	<−6.50
2,4,5-T	0	----	----	----	5	<	<	<−23.6	8	<	2.21	<−2.21	5	<	<	<−0.63

Notes: **^a^** Below the limit of detection (LOD) for a pesticide; **^b^** Concentration data in environmental media at 13 child care centers were previously reported in Morgan *et al*. [18].

In addition, heptachlor was detected >50% in the outdoor air and indoor air samples at both the homes and daycare centers. The OP insecticides, chlorpyrifos and diazinon, were detected ≥50% in the dust, outdoor air, and indoor air samples at both settings. In particular at the homes, the median levels of chlorpyrifos were at least three times greater than the median levels of diazinon in the dust, outdoor air, and indoor air samples. For the PY insecticides, *cis*-permethrin and *trans*-permethrin were both detected ≥50% in the dust and indoor air samples at both locations. Cyfluthrin was detected >40% in only the dust samples in both settings. 2,4-D was the only AC herbicide that was detected >50% in the dust samples in both settings. Of the measured pesticides in the environmental media, the combined isomers of chlordane had the highest 95th percentile concentrations (≥25.0 ng/g) in soil samples at both the homes and daycare centers. For the dust samples, the median levels of the combined isomers of permethrin (>1,400 ng/g) were at least 10 times greater than the median levels for all of the other measured pesticides at both locations. Heptachlor had the highest median concentrations occurring among these pesticides in the indoor air samples (≥5.40 ng/m^3^) in both settings.

Table 5 and Table 6 provide the distributions of the 16 pesticides measured in the personal media at 129 homes and 13 daycare centers in NC. For the hand wipe samples, *α-*chlordane, *γ*-chlordane, chlorpyrifos, *cis-*permethrin, *and trans*-permethrin were detected above >50% in both settings. However, the median levels of *cis/trans*-permethrin were at least five times greater in the hand wipe samples than for all of the other measured pesticides. Chlorpyrifos and 2,4-D were detected above 50% in the solid food samples at the homes, and only chlorpyrifos was detected >50% in the solid food samples at the daycares. Lastly, none of the pesticides were detected often (<19%) in the liquid food samples in either setting.

### 3.3. Estimated Potential Exposures and Potential Intake Doses to Pesticides by Route

The children’s estimated median potential exposures (ng/day) and potential intake doses (ng/kg/day) to the eight frequently detected pesticides through the dietary ingestion, nondietary ingestion, and inhalation routes are presented in Table 7. Also for comparison in Table 7, we have provided the established oral reference doses (RfD’s) and/or inhalation reference concentrations (RfC’s) for these pesticides that are available in the US EPA’s Integrated Risk Management System (IRIS) [25]. The estimated median potential intake doses of the children through the dietary ingestion route were the highest for the combined isomers of permethrin at 4.84 ng/kg/day and for 2,4-D also at 4.84 ng/kg/day. For the nondietary ingestion route, the children had the highest median potential intake dose of 2.39 ng/kg/day to the combined isomers of permethrin which was at least an order of magnitude higher than for the next highest pesticide, chlorpyrifos (0.156 ng/kg/day). In contrast, the children’s estimated median potential intake dose through the inhalation route was the most to heptachlor at 1.71 ng/kg/day, followed by chlorpyrifos at 1.42 ng/kg/day.

**Table 5:** Concentrations of pesticides in personal exposure samples collected from 129 children at their homes in North Carolina.

Pesticide	Hand Wipe (ng/cm^2^)				Solid Food (ng/g)				Liquid Food (ng/mL)			
	%	50th	95th	Range	%	50th	95th	Range	%	50th	95th	Range
*Organochlorine Insecticides*												
Aldrin	1	< **^a^**	<	<−0.02	2	<	<	<−0.47	0	----	----	----
*α*-Chlordane	51	0.004	0.06	<−0.16	17	<	0.15	<−0.47	5	<	<	<−0.04
*γ*-Chlordane	54	0.01	0.09	<−0.17	19	<	0.22	<−0.47	0	----	----	----
*p,p’*-DDT	8	<	0.07	<−0.74	4	<	<	<−2.52	2	<	<	<−0.10
Dieldrin	4	<	<	<−0.21	2	<	<	<−1.58	0	----	----	----
Endrin	3	<	<	<−0.12	1	<	<	<−0.47	0	----	----	----
Heptachlor	22	<	0.04	<−0.15	14	<	0.73	<−1.53	0	----	----	----
Lindane	2	<	<	<−0.01	8	<	0.84	<−12.4	2	<	<	<−0.20
*Organophosphorus Insecticides*												
Chlorpyrifos **^b^**	80	0.02	0.28	<−0.74	65	0.19	2.09	<−19.7	10	<	0.06	<−1.71
Diazinon	46	<	0.08	<−1.55	22	<	0.41	<−6.73	1	<	<	<−0.21
*Pyrethroids Insecticides*												
Cyfluthrin	32	<	0.44	<−0.95	6	<	0.90	<−4.65	0	----	----	----
*cis*-Permethrin	87	0.06	1.46	<−64.0	46	<	15.6	<−80.7	18	<	0.33	<−1.02
*trans*-Permethrin	87	0.05	1.27	<−66.7	46	<	8.7	<−70.4	17	<	0.16	<−0.84
*Acid Herbicides*												
Dicamba	0	----	----	----	16	<	0.88	<−1.67	0	----	----	----
2,4-D **^c^**	9	<	0.02	<−0.04	56	0.35	2.12	<−4.36	2	<	<	<−0.60
2,4,5-T	0	----	----	----	2	<	<	<−1.47	0	----	----	----

Notes: **^a^** Below the limit of detection (LOD) for a pesticide; **^b^** Concentration data in personal media at 129 NC children’s homes were previously reported in Morgan *et al*. [18]; **^c^** Concentration data in personal media were reported for 66 out of 127 NC children’s homes in Morgan *et al*. [20].

**Table 6:** Concentrations of pesticides in personal exposure samples collected from 63 children at their daycare centers in North Carolina.

Pesticide	Hand Wipe (ng/cm^2^)				Solid Food (ng/g)				Liquid Food (ng/mL)			
	%	50th	95th	Range	%	50th	95th	Range	%	50th	95th	Range
*Organochlorine Insecticides*												
Aldrin	3	< **^a^**	<	<−0.17	4	<	<	<−0.17	0	----	----	----
*α*-Chlordane	65	0.01	0.03	<−0.07	13	<	0.11	<−0.33	9	<	0.04	<−0.04
*γ*-Chlordane	65	0.01	0.05	<−0.08	13	<	0.15	<−0.34	0	----	----	----
*p,p’*-DDT	3	<	<	<−0.46	4	<	<	<−1.31	0	----	----	----
Dieldrin	3	<	<	<−0.22	0	----	----	----	0	----	----	----
Endrin	3	<	<	<−0.04	0	----	----	----	0	----	----	----
Heptachlor	23	<	0.05	<−0.05	13	<	0.51	<−0.69	0	----	----	----
Lindane	0	----	----	----	4	<	<	<−0.52	0	----	----	----
*Organophosphorus Insecticides*												
Chlorpyrifos **^b^**	68	0.02	0.07	<−0.08	54	0.10	0.85	<−0.95	14	<	0.06	<−0.15
Diazinon	58	0.01	0.05	<−0.17	25	<	0.17	<−0.89	0	----	----	----
*Pyrethroids Insecticides*												
Cyfluthrin	19	<	0.33	<−0.63	4	<	<	<−5.31	0	----	----	----
*cis*-Permethrin	94	0.07	0.31	<−2.19	25	<	5.17	<−218	14	<	0.06	<−0.55
*trans*-Permethrin	94	0.04	0.26	<−2.13	25	<	2.96	<−149	14	<	0.05	<−0.66
*Acid Herbicides*												
Dicamba	0	----	----	----	4	<	<	<−0.33	0	----	----	----
2,4-D	3	<	<	<−0.02	38	<	1.55	<−2.17	0	----	----	----
2,4,5-T	0	----	----	----	0	----	----	----	0	----	----	----

Notes: **^a^** Below the limit of detection (LOD) for a pesticide; **^b^** Concentration data in media at 13 child care centers were previously reported in Morgan *et al*. [18].

**Table 7:** The preschool children’s estimated median potential exposures and potential intake doses to frequently detected pesticides by exposure route **^a^**.

Pesticide Class ^b^	Pesticide	Potential Exposure (ng/day)			Potential Intake Dose ^c^ (ng/kg/day)			Oral RfD (ng/kg/day)	Inhalation RfC (ng/m^3^/day)
		Dietary	Nondietary	Inhalation	Dietary	Nondietary	Inhalation		
OC	*α*-Chlordane	<	1.60	8.30	<	0.048	0.237	500 **^e^**	700 **^e^**
OC	*γ*-Chlordane	<	2.69	12.7	<	0.083	0.422	500 **^e^**	700 **^e^**
OC	Heptachlor	<	0.915	62.4	<	0.028	1.71	500	---- **^f^**
OP	Chlorpyrifos **^d^**	81.1	5.16	47.2	2.5	0.156	1.42	----	----
OP	Diazinon	<	0.984	16.9	<	0.03	0.507	----	----
PY	*cis*-Permethrin	84.7	48.1	4.64	2.63	1.39	0.137	50,000 ^e^	----
PY	*trans*-Permethrin	74.5	35.4	2.73	2.21	1.00	0.088	50,000 ^e^	----
AC	2,4-D	188	1.45	4.00	4.84	0.042	0.099	10,000	----

Notes: **^a^** Estimated for pesticides that had ≥45% detects in two or more sampled media; **^b^** Pesticide classes include organochlorine insecticides (OC), organophosphorus insecticides (OP), pyrethroid insecticides (PY), and AC herbicides; **^c^** Assuming a 50% absorption for a pesticide for each route of exposure; **^d^** Data were calculated from Morgan *et al*. [18]; **^e^** Value equals total chlordane or total permethrin (not individual isomers); **^f^** No oral reference dose (RfD) or inhalation reference concentration (RfC) was available in the US EPA’s Integrated Risk Information System (IRIS) [25].

### 3.4. Estimated Potential Aggregate Exposures and Potential Aggregate Intake Doses to Pesticides

**Figure 1:**
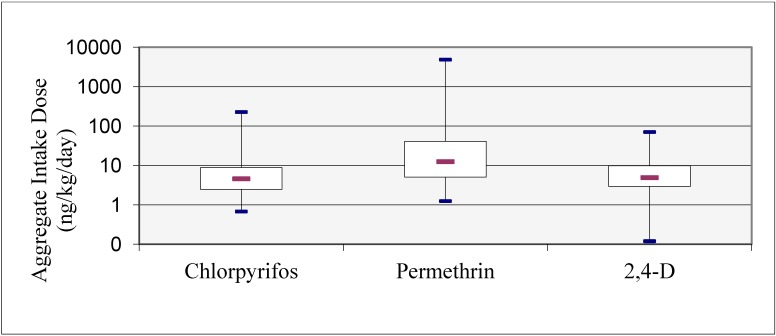
The children’s estimated potential aggregate intake doses to chlorpyrifos, permethrin, and 2,4-D **^a,b^**.

Notes: **^a^** Combined *cis*- and *trans-*isomers; **^b^** The line within each box represent the median concentration value for a pesticide.

The children’s estimated potential aggregate intake doses by all three exposure routes were quantifiable for chlorpyrifos, *cis/trans*-permethrin, and 2,4-D and are depicted as a box-and-whiskers plot in Figure 1. The estimated median potential aggregate intake doses of the children were 4.6 ng/kg/day for chlorpyrifos, 12.5 ng/kg/day for *cis/trans*-permethrin, and 4.9 ng/kg/day for 2,4-D. At the 95th percentile, the children’s estimated potential aggregate intake doses were 31.7 ng/kg/day (chlorpyrifos), 397 ng/kg/day (*cis/trans*-permethrin), and 22.5 ng/kg/day (2,4-D). The results show that dietary ingestion (>60%) was the predominant route of the children’s exposures to all three pesticides.

## 4. Discussion

As limited published data exist on the absorption rates of many pesticides in humans, scientists must frequently rely on default assumption values to help calculate the estimated potential intake doses of children to pesticides by exposure route. A common approach is to use the most conservative absorption rate value of 100% for a pesticide for a child by exposure route (inhalation and ingestion) [15,26,27]. This approach assumes that 100% of the total amount of the pesticide, after exposure, is absorbed into the body [26]. However in recent years, research has shown that pesticide absorption rates in humans can vary greatly by such things as class of pesticide, exposure route, and administered vehicle (e.g., corn oil), and these absorption rates have been generally substantially less than 100% [28,29,30,31]. Therefore in the CTEPP study, we selected a more reasonable default absorption rate of 50% for a pesticide by each exposure route [24]. Appendix Table A1 illustrates the differences in the maximum potential intake doses for the CTEPP children by route when using the default absorption rate of 100% *versus* 50%. For example, the maximum potential dietary intake dose of one CTEPP child to permethrin is twice the amount when using a 100% absorption rate (9,700 ng/kg/day) compared to using our 50% absorption rate (4,850 ng/kg/day). More research is needed to quantify the actual absorption rates of pesticides by route in humans (e.g., *in vitro* assays) which would greatly improve pesticide exposure assessments for children.

In this current work, the results show that of the measured OC insecticides only *α-*chlordane, *γ-*chlordane, and heptachlor were detected ≥50% in several different media at the preschool children’s homes and daycare centers in NC. Inhalation of indoor air and outdoor air was found to be the predominant exposure route of the children to both *α/γ-*chlordane (~83%) and heptachlor (~98%). An interesting observation was that the NC CTEPP preschool children had the highest estimated median potential inhalation dose of 1.71 ng/kg/day to heptachlor (maximum value = 118 ng/kg/day) compared to all of the other pesticides measured in this study. This finding is supported by research conducted by Wilson *et al*. [14,15] showing that inhalation was a major exposure route of nine preschool children to eight different OC insecticides at their homes and daycare centers in NC in 1997, and heptachlor substantially contributed to their OC insecticide exposure by this route. This is a concern as almost all uses of heptachlor were phased-out by the late 1980’s, except to control fire ants in subsurface electrical power transformers and cable boxes, because of its persistence in the environment and in the body [1]. In addition, an established RfC for heptachlor is currently not available in the U.S. EPA’s IRIS [25], therefore, we could not ascertain if the children’s potential inhalation doses were below a level of concern in these environments. Because heptachlor is persistent and bioaccumulative in the body, more research is needed to understand children’s temporal exposures to heptachlor and potential health risks in places where children frequently spend their time (*i.e*., residences, daycares, schools, and parks) [1]. 

At the time that the CTEPP study was conducted in 2000–2001, the OP insecticides, chlorpyrifos and diazinon, and the PY insecticides, permethrin and cyfluthrin, were commonly used to control insect pests at dwellings and on agricultural crops. Our results show that these insecticides, except for cyfluthrin, were detected ≥50% in several different media at the children’s homes and daycare centers. Of these insecticides, the CTEPP children had the highest estimated median potential aggregate intake doses to the combined isomers of permethrin (12.5 ng/kg/day), followed by chlorpyrifos (4.6 ng/kg/day). Dietary ingestion was the predominant route of the children’s exposures to both permethrin (~65%) and chlorpyrifos (~61%). In comparison, Morgan *et al*. [19,23] have reported about three times lower estimated median potential aggregate intake dose (4.0 ng/kg/day) to the combined isomers of permethrin for 111 preschool children from the OH component of the CTEPP study; dietary ingestion (~60%) also contributed the most to their exposure. In another study conducted in 2001 by Tulve *et al*. [32,33], they showed that permethrin was frequently detected (>50%) in several media at nine preschool children’s homes in Florida that reported frequently using products containing pesticides. The authors reported that both dermal (57%) and dietary ingestion (33%) likely contributed substantially to the children’s cumulative exposures (nmol/day; *not intake dose*) to pyrethroids (which included permethrin), however, they state that the results are limited due to the small sample size of children [33]. These above studies suggest that there are likely geographic differences in the use and amount of permethrin applied in residential settings in the U.S. and more research is needed. For chlorpyrifos, our results (4.6 ng/kg/day) were about six times lower than the results reported in Wilson *et al*. [15] having estimated median potential aggregate exposures of 30.0 ng/kg/day for nine preschool children at their homes and daycare centers in NC in 1997. In contrast, our study results are only about two times lower than the results reported in Clayton *et al*. [34] showing a median aggregate intake dose to chlorpyrifos of 11.7 ng/kg/day for 56 children, ages 3–12 years old, at their homes in Minnesota in 1997. In the more recent Pesticide Exposures of Preschool Children Over Time (PEPCOT) study conducted between 2003–2005 [27], the authors reported estimated median potential aggregate intake doses of 8.0, 6.2, and 6.2 ng/kg/day to chlorpyrifos (*assuming a 100% absorption rate*) for 50 preschool children (older sibling) at their homes in NC in 2003, 2004, and 2005, respectively. The CTEPP children’s estimated median potential aggregate intake doses to chlorpyrifos are slightly higher than for the PEPCOT children when assuming a 100% default absorption rate for a pesticide. Overall, these above studies suggest that preschool children’s exposures to chlorpyrifos are declining over the last decade in the U.S. and are likely associated with the U.S. EPA’s 2001 phase-out of this insecticide [7,27]. This information is supported by Clune *et al*. [35] that showed a substantial decline in the last decade in urinary dialkylphosphate (DAP) levels of OP insecticides in over 3,000 adults from the U.S. National Health and Nutrition Examination Survey (NHANES III [1988–1994] and NHANES 1999–2004). The authors suggest that the lower DAP levels appear to be related to the U.S. EPA phase-out of chlorpyrifos and diazinon at residences and similar settings [35]. 

Among the measured AC herbicides in our study, only 2,4-D was detected above 50% in any medium at the children’s homes and daycare centers. The CTEPP children’s estimated median potential aggregate intake dose to 2,4-D was 4.9 ng/kg/day, and dietary ingestion accounted for almost all (~97%) of their exposure. The children’s estimated maximum potential aggregate intake dose of 70.8 ng/kg/day (*data not shown*) was at least 140 times lower than the RfD of 10,000 ng/kg/day in the U.S. EPA’s IRIS [25]. Wilson *et al*. [15] reported a much higher estimated median potential aggregate intake dose of 87.6 ng/kg/day to 2,4-D for nine preschool children at their homes and daycare centers in 1997. In a different study, Nishioka *et al*. [36] reported that dietary ingestion (94%) was also the predominant route of young children’s exposures to 2,4-D before application of this insecticide at seven Midwestern homes. However after application of 2,4-D, dietary ingestion (53%) and nondietary (41%) ingestion both became important routes of the children’s exposures to this insecticide at home [36]. For the more recent PEPCOT study [27], the children’s estimated potential median aggregate intake doses to 2,4-D ranged from 8.2–13.49 ng/kg/day between 2003–2005, and dietary ingestion (88%) was the predominant exposure route. The above studies suggest that dietary ingestion was the predominant route of these preschool children exposures to 2,4-D between 1997 and 2005 in NC. However, it remains unclear which consumed foods likely contributed to the CTEPP children’s dietary exposures to 2,4-D as solid and liquid food samples were separately consolidated over a 48-h monitoring period. Furthermore in a recent article by Morgan and Jones [37], the authors did not find any association between the reported weekly intake frequency of 65 different food items and mean urinary 2,4-D concentrations in 135 CTEPP children from NC and OH. More research is needed to quantify the levels of 2,4-D and other pesticides in individual food items consumed by young children as few data exist in the literature. 

## 5. Conclusions

In conclusion, the CTEPP preschool children were concurrently exposed at low levels to a number of past-use and current-use pesticides from several sources and routes of exposure at their homes and daycare centers in NC. Pesticides that were detected ≥50% in several different media at these locations included *α*-chlordane, *γ*-chlordane, heptachlor, chlorpyrifos, diazinon, *cis*-permethrin, *trans*-permethrin, and 2,4-D. However, the children’s exposures to these eight pesticides varied greatly by exposure route. Inhalation was the predominant route of the children’s exposure to *α/γ* chlordane (~83%), heptachlor (~98%), and diazinon (~94%) and to a lesser extent to chlorpyrifos (~35%). Dietary ingestion was the major exposure route of the children to chlorpyrifos (~61%), *cis/trans*-permethrin (~65%), and 2,4-D (~97%). Lastly, nondietary ingestion was also an important secondary exposure route to *cis/trans*-permethrin (~32%). 

## Appendix

**Table A1:** The maximum potential intake dose of a CTEPP child for a pesticide by route when zsing either a 50% or 100% default absorption rate value **^a^**.

Class ^b^	Pesticide	Maximum Potential Intake Dose at a 50% Absorption Rate (ng/kg/day)			Maximum Potential Intake Dose at a 100% Absorption Rate (ng/kg/day)		
		Dietary	Nondietary	Inhalation	Dietary	Nondietary	Inhalation
OC	*α*-Chlordane	10.1	1.51	9.37	20.2	3.02	18.7
OC	*γ*-Chlordane	8.64	1.71	13.3	17.3	3.42	26.6
OC	Heptachlor	15.9	6.17	118	31.8	12.3	236
OP	Chlorpyrifos **^c^**	217	5.84	53.1	434	11.7	106
OP	Diazinon	40.2	16.7	380	80.4	33.4	760
PY	*cis*-Permethrin	2,850	143	6.84	5,700	286	13.7
PY	*trans*-Permethrin	2,000	151	8.38	4,000	302	16.8
AC	2,4-D	60.6	10.1	1.04	121	20.2	2.08

Notes: **^a^** Estimated for pesticides that had ≥45% detects in two or more sampled media; **^b^** Pesticide classes include organochlorine insecticides (OC), organophosphorus insecticides (OP), pyrethroid insecticides (PY), and AC herbicides; AC); **^c^** These data were calculated from Morgan *et al*. [18].
